# Endoscopic argon plasma coagulation treatment for late dumping syndrome in patients with Roux-en-Y gastric bypass

**DOI:** 10.3389/fendo.2025.1662911

**Published:** 2025-08-25

**Authors:** Sara Mera-Carreiro, Blanca Bernaldo-Madrid, Clara Rodríguez-Carrillo, José Miguel Esteban-López-Jamar, Clara Marcuello-Foncillas, Natalia Pérez-Ferre, Ana Ramos-Leví, Pilar Matía-Martín, Miguel Ángel Rubio-Herrera

**Affiliations:** ^1^ Department of Endocrinology and Nutrition, Hospital Clínico San Carlos Instituto de Investigación Sanitaria del Hospital Clínico San Carlos (IdISSC), Madrid, Spain; ^2^ Department of Endocrinology and Nutrition, Hospital HM Madrid Río, Instituto de Investigación Sanitaria HM Hospitales, Madrid, Spain; ^3^ Department of Gastroenterology and Hepatology, Hospital Clínico San Carlos Instituto de Investigación Sanitaria del Hospital Clínico San Carlos (IdISSC), Madrid, Spain; ^4^ Department of Medicine, Faculty of Medicine, Universidad Complutense, Madrid, Spain

**Keywords:** endoscopic argon plasma coagulation, dumping syndrome, post-bariatric hypoglycemia, postprandial hypoglycemia, Roux-en-Y gastric bypass, weight regain

## Abstract

**Introduction:**

Dumping syndrome (DS) and postprandial hypoglycemia (PPH) are challenging complications encountered after Roux-en-Y gastric bypass (RYGB). Surgical revision is often the next therapeutic step when pharmacological and dietary treatments fail to control DS and PPH. Endoscopic argon plasma coagulation (APC) is a less invasive alternative that reduces the diameter of the gastrojejunal anastomosis (GJA). The aim of the study is to evaluate the efficacy and safety of APC in managing postprandial hypoglycemia (PPH) after RYGB.

**Methods:**

This retrospective study included patients who underwent endoscopic APC for GJA reduction between 2018 and 2022. Improvement of PPH, and anthropometric data were evaluated.

**Results:**

Twenty-five patients aged 52.3 ± 9.2 years, with PPH and poor response to pharmacological treatment, were recruited. All patients had an average of two APC endoscopic procedures (range 1–4), initial GJA diameter of 26.8 ± 7.2 mm, and post-APC diameter of 16.4 ± 4.4 mm. Adverse events were mild and did not require hospitalization. Symptoms improved in 100% of patients with a decrease in Sigstad score from 8.2 ± 1.9 to 0.9 ± 2 (p < 0.0001) and resolution of PPH (p < 0.0001) over the 24-month follow-up. Of these, 84% discontinued pharmacological treatment. In addition, 60% of the patients who regained weight from the nadir after RYGB had a significant percentage of total body weight loss (% TBW) (p < 0.01) after APC during the 2-year follow-up.

**Conclusion:**

APC is effective, safe, and reproducible in managing PPH in patients who undergo RYGB, refractory to dietary and pharmacological treatments. It also contributes to weight loss after weight regain.

## 1 Introduction

The number of bariatric surgeries is increasing owing to the high prevalence of obesity. Roux-en-Y gastric bypass (RYGB) is one of the most common bariatric surgeries worldwide, with over 200,000 procedures performed in 2018 (1). These surgeries may lead to complications, such as vitamin or iron deficiency, protein and calorie malnutrition, leakage, hernia, and dumping syndrome (2). Dumping syndrome is a common consequence of this procedure, and its reported incidence varies widely (12–76.9%) in the literature (3, 4).

Dumping symptoms may occur within an hour of a meal (early dumping) or up to 3 hours after a meal (late dumping). These symptoms consist of a combination of gastrointestinal and vasomotor symptoms, including tachycardia, fatigue, flushing, sweating, syncope, and rarely shock and seizures caused by profound hypoglycemia. Early dumping syndrome results from rapid pouch emptying of the hyperosmolar contents into the small intestine, causing osmotic fluid to shift from the blood into the intestinal lumen. Late symptoms result from exaggerated incretin responses due to early nutrient delivery to the distal small intestine, causing the symptoms previously described, including reactive hypoglycemia, referred to as postprandial hypoglycemia (PPH), which usually occurs no earlier than 6 months after surgery (5, 6). The pathophysiological mechanisms of PPH are unclear. Proposed hypotheses include increased incretin secretion, dysregulation of the counterregulatory response to hypoglycemia, or anatomical alterations such as dilation of the gastrojejunal anastomosis and accelerated gastric emptying. In most cases, both early and late dumping syndrome is temporary and improves within the first 1–2 years after surgery (7). Dietary modifications, followed by pharmacological treatments, such as acarbose, canagliflozin, calcium antagonists, or somatostatin analogs, can be used as needed to control hypoglycemia symptoms. In patients who do not respond to pharmacological treatment, the value of continuous enteral feeding and surgical re-intervention has had variable results, and a conservative, nonsurgical approach is recommended (8). Morphologically, dumping syndrome usually correlates with dilatation of the gastrojejunal (GJ). Thus, endoscopic tightening of the anastomosis results in delayed gastric emptying and symptomatic improvement (9). This can be achieved using endoscopic suturing, ablation, or sclerotherapy. Argon plasma coagulation (APC) is a noncontact electrocoagulation method, and reliable data support the similarity between APC alone and endoscopic suturing in reducing anastomosis size (10, 11). Some studies have demonstrated the effectiveness of APC plus endoscopic suturing (12, 13), although no studies on APC treatment alone for PPH symptoms have been conducted. This series highlights our experience using only endoscopic APC to treat dumping syndrome with PPH after RYGB, and we also evaluate its efficacy and safety.

## 2 Materials and methods

### 2.1 Study design and patients

A retrospective single-arm study was conducted for five year period of 2018–2022 in a tertiary care center on patients with dumping symptoms and confirmed hypoglycemia. This study was approved by the local ethics committee (C.I. 24/011-E), and informed consent was obtained from all participants according to the principles of the Declaration of Helsinki.

Adults with prior RYGB for obesity with late dumping symptoms, suggestive of postprandial hypoglycemia with clinical evidence of Whipple’s triad and proven hypoglycemia (< 54 mg/dL)after a capillary blood sample finger prick test and/or during a mixed meal tolerance test of 240 minutes were included. All participants were treated with dietary modifications and pharmacological therapy in a stepwise manner. Our standard treatment regimen usually begins with acarbose 100 mg every 8 hours. If there is no improvement and/or the patient experienced digestive intolerance, canagliflozin 100 mg every 8 hours and/or verapamil 120 mg every 24 hours were added. Exceptionally, when a patient is intolerant to the previous medication or it is clinically ineffective, treatment with subcutaneous octreotide, 100 ug every 8–12 hours, is suggested. Patients with weekly persistent postprandial hypoglycemia, for at least 3-month period, were eligible for APC when the size of the GJ anastomosis was greater than 15 mm. After the APC sessions, all medications were stopped progressively. Other alternative treatment methods to APC treatment (such as endoscopic suturing or surgical revision) were offered to patients.

We collected data from the patients’ clinical records, including age, sex, body mass index (BMI) before bariatric surgery and at the time of the endoscopic procedures, time since bariatric surgery, and percentage of weight loss after APC. The severity of dumping symptoms was assessed using the Sigstad score (14).

### 2.2 Endoscopic intervention

**Figure 1:**
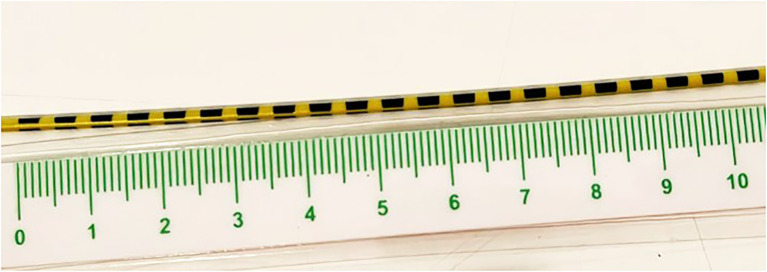
Calibration method used for gastrojejunal anastomosis, based on a millimeter-sized pediatric probe (see methods).

All patients were admitted to the endoscopy unit after a 12-hour fast. They were placed in the left lateral decubitus position, sedated with propofol under the supervision of an anesthesiologist, and were not intubated. The endoscopist accessed the stomach pouch and gastrojejunostomy using a 180 cm diagnostic gastroscope. The diameter size of the GJ anastomosis was measured using a calibrated pediatric probe with millimeter accuracy (from a single-use biopsy forceps [Radial Jaw™ 4, Boston Scientific] (**Figure 1**). before and prior to the following the procedure. Once the ERBE USA Argon Plasma Coagulator Circumferential Probe (O.D. 2.3 mm/6.9 Fr, length 220 cm) was located at the gastrojejunostomy, it was connected to a generator set to pulsating 1.9 L/min and 90 W, and argon was delivered circumferentially at the gastrojejunostomy. We try to treat between 6 and 10 mm of thickness around the entire circumference of the anastomosis. The procedure was performed by a single operator and took approximately 5–10 minutes, after which the patient was transferred to the recovery area. The hospital stay duration was approximately 30–60 min.

All patients were discharged on the same day with instructions regarding a liquid diet for 7 days, following by a soft diet for 10–15 days, proton pump inhibitor therapy (esomeprazole 40 mg every 12 hours for two weeks, and then every 24 hours thereafter; antiemetics (ondansetron 4 mg, if necessary), and pain medications (alternate between paracetamol 650 mg every 8 hours and metamizole 525 mg every 8 hours, when needed). Patients underwent between 1 and 4 consecutive APC endoscopic sessions depending on the final anastomotic diameter, improvement of hypoglycemic symptoms. Patients were followed up monthly for 6 months after the procedure and every 3–6 months thereafter. Follow-up visits included weight measurement, clinical history, and examination.

### 2.3 Study outcomes

All patients were followed up at an outpatient clinic. Postprocedural symptoms were assessed using the Sigstad score. Remission of PPH takes into account the disappearance of Whipple’s triad: absence of clinical symptoms of hypoglycemia, in the context of a normal postbariatric diet (without simple carbohydrates), along with daily pre- and postprandial capillary blood glucose measurements for each main meal containing carbohydrates. Blood glucose measurements are also performed in the presence of any known symptoms of hypoglycemia. This practice is performed systematically for at least 6 months after completing all endoscopies, with measurements being spaced out over time. Early and late endoscopic complications were assessed. The clinical symptoms of postprandial hypoglycemia, as well as the evolution of weight, were monitored over 24 months.

### 2.4 Statistical analysis

Continuous variables are summarized as mean and standard deviation or median with a range interval, whereas categorical variables are expressed as percentages. Continuous variables were compared using the independent sample t-test. For variables with a skewed distribution, the Mann–Whitney U test was used for mean comparisons. The chi-square test was used to analyze categorical data. All statistical analyses were performed using the Statistical Package for Social Sciences software (IBM SPSS version 26.0 and JASP version 0.18.1, 2023).

## 3 Results

### 3.1 Descriptive results

Twenty-five patients with PPH and refractory to pharmacological treatment underwent endoscopic APC for dumping syndrome after RYGB. The mean patient age was 52.3 ± 9.2 years, and 80% were women. Pre-RYGB weight and BMI were 113.2 ± 17.2 kg and 42.7 ± 5.5 kg/m2, respectively. The nadir BMI was 26.6 ± 3.8 kg/m2, corresponding to a 37.1 ± 10.2 percent total body weight loss following gastric bypass.

Dumping symptoms appeared 26 (20–84) months after the surgery. Between the minimum weight achieved after bariatric surgery and the endoscopic procedure (> 24 months), 15/25 patients (60%) had ≥ 10% weight regain (WR), whereas 10/25 patients (40%) maintained their total body weight despite the presence of PPH symptoms (increased body weight 19.3 ± 4.6% vs. 1.5 ± 4.6% from nadir; p < 0.01). No patient had diabetes mellitus nor other major comorbidities before endoscopic treatment.

At the time of intervention, the mean weight and BMI were 82.0 ± 19.5 kg and 30.8 ± 6.25 kg/m2, respectively. Most patients had received dietary instructions (fractionated food and low-carbohydrate intake) and pharmacological treatment (22 patients (88%) received acarbose and/or canagliflozin, 2 patients (8%) received calcium antagonists together with either of the two previous drugs, and 1 patient (4%) received octreotide)).

### 3.2 Endoscopic procedures

**Figure 2:**
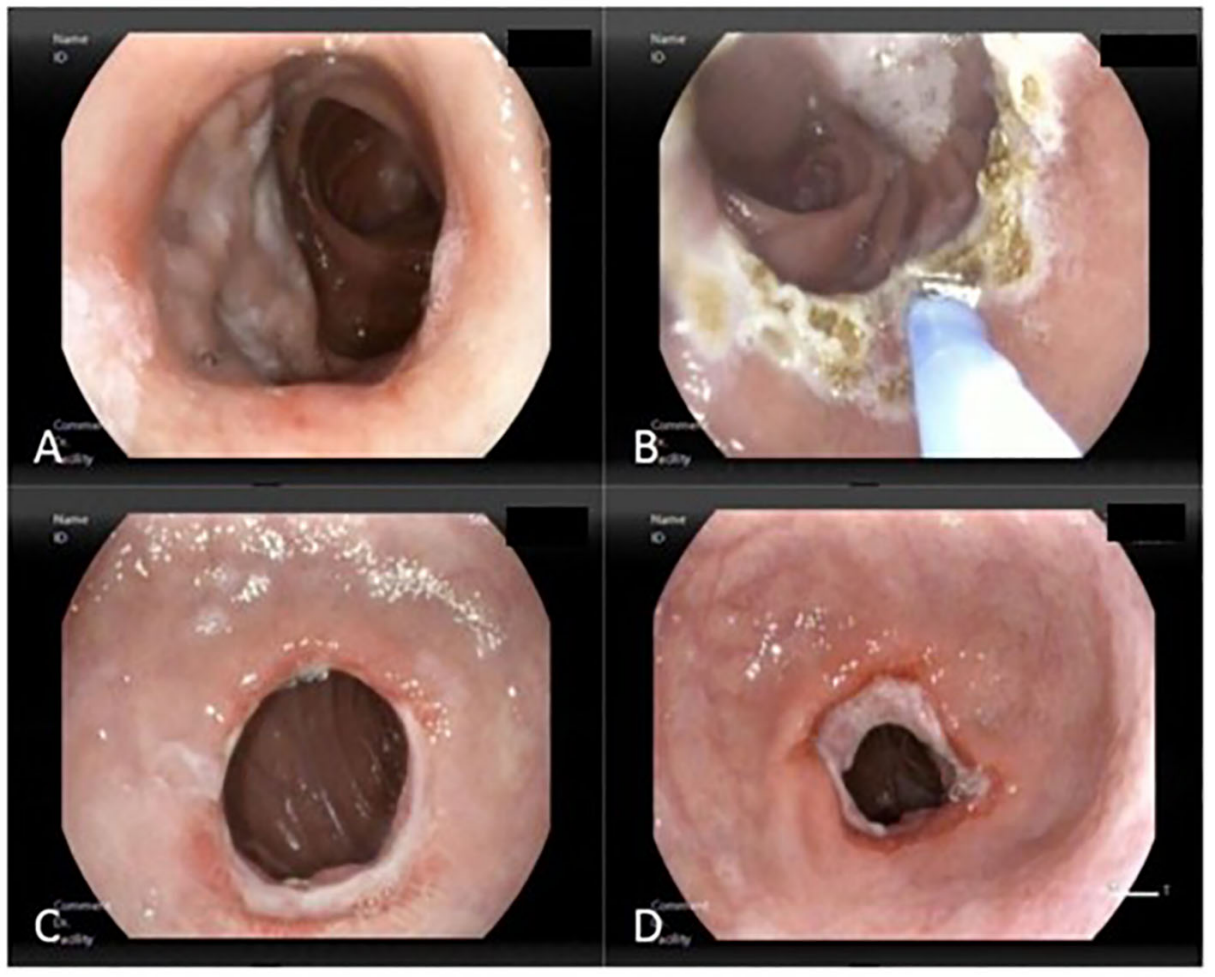
Reduction of the gastrojejunostomy (GJ) using high-flow APC (1.9L/min and 90W). **(A)** Dilated GJ of 20 mm in diameter, **(B)** APC of the entire circumference, **(C)** Ulceration of the circumference due to previous APC, **(D)** Reduction of the GJ to 10 mm.

**Figure 3:**
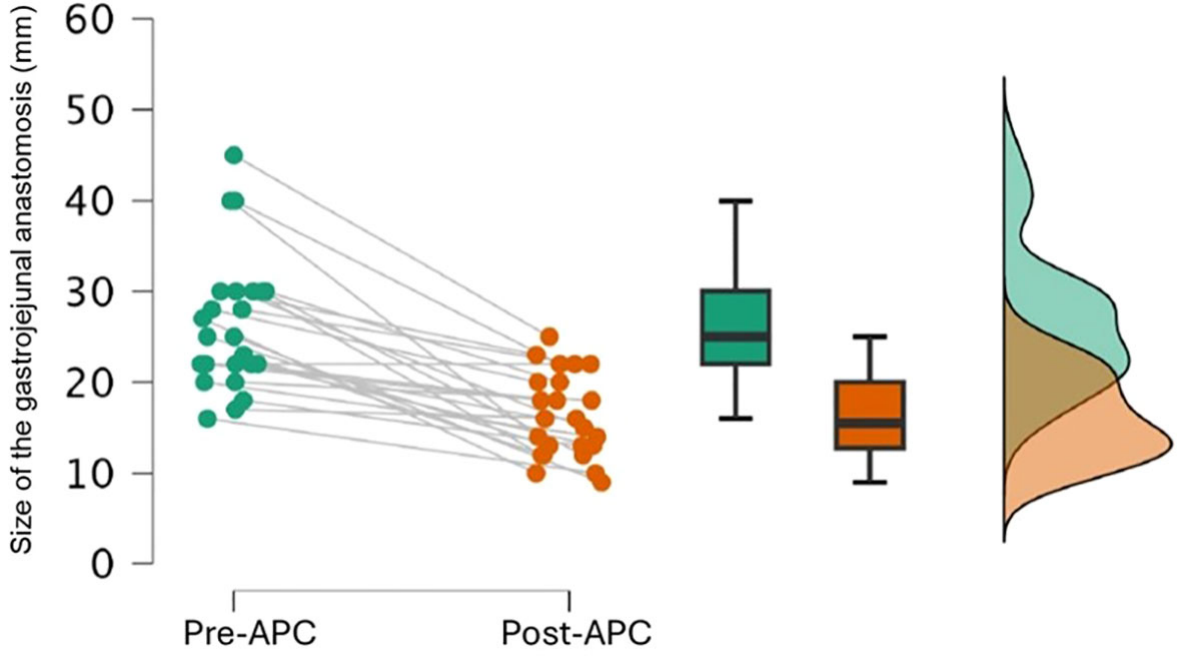
Changes in the diameter of the GY anastomosis after APC.

All patients underwent an average of two endoscopic APC procedures (range 1–4), with an average of 4 weeks between each procedure. If an active ulcer was present from the previous APC session, the upcoming APC procedure was postponed for other 4 weeks. The aim was to achieve a sufficient reduction in the diameter of the anastomosis to resolve hypoglycemia and late dumping symptoms (**Figure 2**). Before the procedure, the mean GJ anastomosis diameter was 26.8 ± 7.2 mm and was reduced to a final diameter of 16.4 ± 4.4 mm (**Figure 3**). A total of 11/25 patients (44%) achieved an anastomosis diameter ≤ 15 mm. Clavien-Dindo Grade 1 complications were reported and did not required hospitalization (15). Consisted of pain (three patients, 12%), transient vomiting (two patients, 8%), minor bleeding (one patient, 4%) and reduction of the GJ diameter <10 mm (three patients, 12%). No patients require hospitalization.

### 3.3 Clinical outcomes

The 12-month follow-up rate was 100%, and 88% at 24 months (one patient changed residence and two patients were lost to follow-up in the second year). Symptoms improved 100% of patients with a decrease in Sigstad score from 8.2 ± 1.9 before APC to 0.9 ± 2 points (p < 0.0001) 12 months after APC. Of these patients, 84% were able to discontinue pharmacological treatment during the 24 months of follow-up. In addition, all patients who regained weight (12/25patients) had significant mean total body weight loss (% TBW) after APC (p < 0.01). The average weight loss was 11.3 ± 3.9% of TBW between 12 and 24 months.

**Figure 4:**
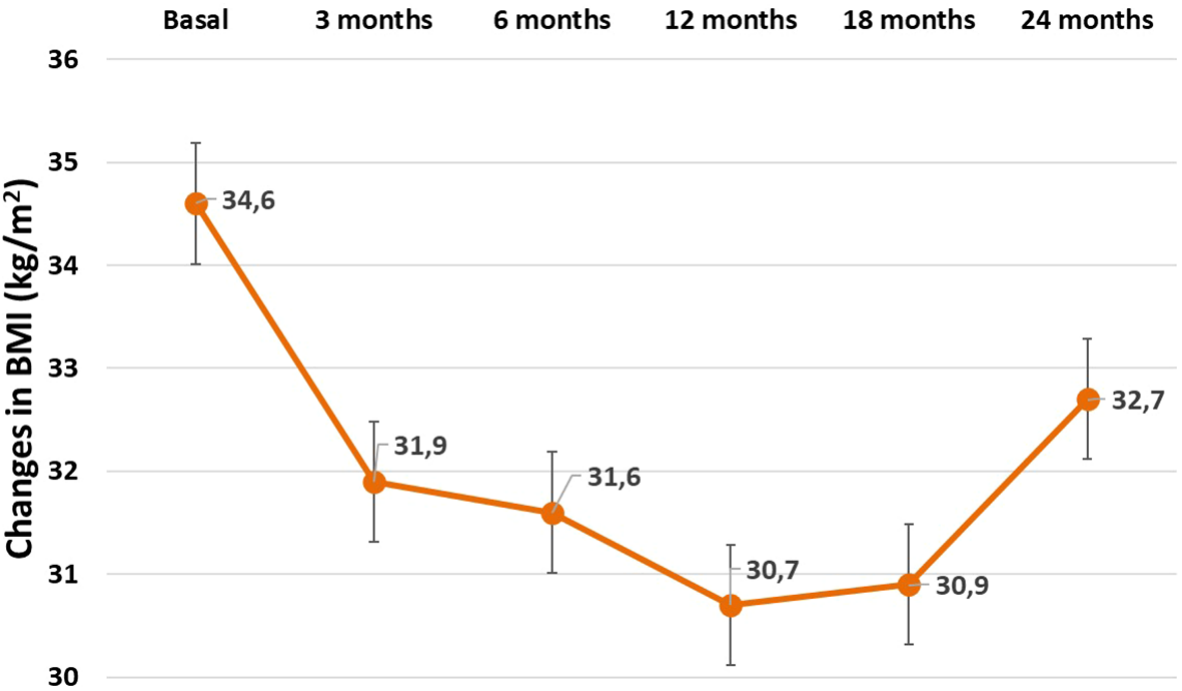
Changes in the Body mass index (BMI) after APC procedure in patients experiencing previous weight regain after bariatric surgery.

Patients with WR after surgery had a BMI of 34.6 ± 4.9 kg/m2 before endoscopic treatment, which decreased to a minimum of 30.5 ± 4.3 kg/m2 after treatment with APC. After 18 months of follow-up, a moderate weight rebound was observed (**Figure 4**), without resulting in an increase in symptoms of PPH. Patients without WR after RYGB maintained their BMI throughout the 24 months of follow-up. They had a BMI of 25.4 ± 4.2 kg/m2 before APC and a minimum BMI of 25.2 ± 3.7 kg/m2 after the treatment. In this group the median weight loss after APC was only 1–2 kg.

No association was found between the change in the diameter of the GJ anastomosis after APC and the percentage of weight lost. Even the association between a cut-off point for a diameter less than 15 mm and the percentage of weight loss showed no significant statistical differences.

## 4 Discussion

RYGB remains one of the most common bariatric surgeries worldwide; as a result, postprandial hypoglycemia have been a cause of concern (14, 16). No consensus exists on how to effectively diagnose PPH (17). Despite certain limitations, combining provocation tests, such as the mixed meal test and/or continuous glucose monitoring, can improve diagnostic performance (18). Some authors define PPH when repeated blood glucose levels below 54 mg/dL are associated with hypoglycemia symptoms (19). In addition to reporting PPH symptoms according to the Whipple criteria, we confirmed the diagnosis in some patients using the mixed meal test for 240 min to capture all late hypoglycemia. Alternatively, the Sigstad dumping score is the most popular questionnaire for evaluating dumping symptoms, even though it is not the most specific. The questionnaire was developed and validated for complaints of early dumping after partial gastrectomy, so the accuracy for late dumping after bariatric surgery has not been established (8, 14). After the APC procedure, the Sigstad score in our patients decreased significantly (approximately 90%) for at least 1 year, coinciding with clinical improvement and the and the absence of hypoglycemia evidenced with a glucometer.

A consensus exists that gastric surgery can reduce gastric volume and remove the barrier function of the pylorus, which allows rapid delivery of food into the small intestine (8). Accelerated gastric emptying of nutrients to the small intestine through a dilated GJ anastomosis is a characteristic feature and critical event in the pathogenesis of dumping syndrome and PBH. Glycemic index and the quantity of carbohydrates ingested, enteric autonomic system dysfunction alongside altered islet cell function, dysregulation of the counterregulatory response to hypoglycemia, disordered serotonin metabolism and changes in energy metabolism can also exacerbate hypoglycemia in susceptible individuals (5, 20). Initial management focuses on dietary modification, emphasizing smaller and more frequent meals while avoiding rapidly absorbed simple sugars. Pharmacological interventions are the next step (21). Acarbose can delay carbohydrate digestion inhibiting intestinal α-glucosidases (22) and canaglifozin acts by inhibiting sodium-glucose cotransporter 1 receptors in the gut (23). Somatostatin analogs are also highly effective for managing postprandial hypoglycemia but are not tolerated by all patients because of gastrointestinal side effects (24) and, is difficult to access due to the high cost. Usually, most patients can adequately control their hypoglycemic symptoms after all these measures. However, a small percentage of patients can experience intractable PPH, which significantly affects their quality of life and requires surgery or novel endoscopic treatments. Although the 25 patients in our series may appear to be a small cohort.we included those who persisted with at least one episode of severe hypoglycemia per week for more than 3 months, despite prescribed dietary and pharmacological treatment during the 5-year observation period. A limited number of patients have been reported in previous series for different endoscopic modalities (12, 25, 26) because severe PPH with neuroglycopenic symptoms represents less than 1–2% of all patients who have undergone RYGB in our health environment (27) and only a small percentage of these patients require endoscopic treatment (28). Guidelines recommend that conservative treatment should be the preferred first-line approach, with surgery being considered as a last resort (19). The introduction of endoscopic therapies, such as APC, further avoid the need for surgical reintervention, as can be seen in our series. None of our patients required surgical revision, even beyond the two years of follow-up of this study. Several studies have reported surgical re-intervention, such as bypass reversal, interposed bowel loops with variable outcomes (8). While primary RYGB has an acceptably low complication rate, secondary RYGB and laparoscopic pouch revisions are associated with higher morbidity (29–36).

Endoscopic approaches have emerged as promising alternatives. The feasibility of endoscopic suturing to reduce the size of the GJ anastomosis was first demonstrated in patients with WR after RYGB (37). Subsequently, a pilot study using a superficial thickness plication device was reported (38). However, over the years, full-thickness suturing devices, with or without their combination with APC, have replaced superficial thickness plication devices because of their better results (10–13). When treating at a higher intensity (over 70 Watts) and at a higher flow rate (from 1.5 to 1.9 liters), the damage produced sometimes reaches the submucosa and the healing process leads to fibrosis, which contributes to reducing the diameter of the gastrojejunal anastomosis. A recent meta-analysis showed that both endoscopic full-thickness suturing plus argon plasma mucosal coagulation and APC alone provided significant and comparable anastomotic reduction results with a good safety profile. APC alone typically requires more than one endoscopic session but has the advantage of being less technically demanding and more universally available (11).

The concept of endoscopic tightening of the dilated GJ anastomosis for treating dumping syndrome emerged several years ago with promising results (39). However, to date, no study of APC alone for treating PPH has been conducted. In our study, we demonstrate that reducing the GJ anastomosis diameter with endoscopic APC effectively induces remission of PPH in 84% of the patients refractory to pharmacological therapy. Patients required an average of two APC sessions (range 1–4) to control PPH symptoms during a 2-year follow-up. The procedure is quick, safe, and reliable, with few complications, and can be repeated if symptoms persist. In most cases, the diameter of the gastrojejunostomy was not assessed at the end of the follow-up period because this was a retrospective clinical study, and if the patient was asymptomatic, there was no need to repeat the APC procedure or endoscopy. This was due to the cost and inconvenience to patients, and because it has been verified that the final diameter after APC does not statistically correlate with clinical improvement or weight loss in our series.

In a few cases, described in other series, if the reduction of the GJ anastomosis with APC was insufficient to improve symptoms;, patients could be offered the option of full-thickness suturing (11, 13, 39),. In our study all cases improved, but if they did not, or if in the follow up symptoms recurred, and repeated APC is insufficient, suturing could be an option. Full-thickness suturing of the GJ anastomosis is more appropriate for weight loss as it can make the reduction of the anastomosis more permanent. However, our aim was to improve the symptoms of HPP; therefore, APC alone may be sufficient with fewer complications. The increase in the diameter of the GJ anastomosis and the gastric pouch was not only a cause of PPH but also a significant cause of WR. In our study, 15/25 patients (60%) had ≥ 10% WR, but 10/25 patients (40%) maintained their weight. Although weight loss after APC was not the aim of our study, the reduction in the diameter of the GJ anastomosis undoubtedly favored additional weight loss in patients who regained weight. In contrast, patients without WR maintained their BMI throughout the 24 months of follow-up. Maximum weight loss was achieved at a median of 12–18 months after the endoscopic procedure (approximately 11% body weight), while partial weight recovery was observed at 24 months of follow-up. These data were similar to those obtained in a series of patients with WR who underwent APC (40, 41). In this study, we did not find a statistically significant association between the final diameter of the GJ anastomosis and the percentage of weight loss achieved. We clinically observed a restriction in food intake, after each procedure, owing to the inability to ingest large amounts of food and the increase in satiety. The previous arguments can explain the weight loss during the first year when the sessions were performed.

The limitations of this study include its unicentric and retrospective design. However, close follow-up of this specific group over a long period has allowed us to obtain valuable clinical results that may help enhance nonsurgical therapeutic options for patients with PPH.

To the best of our knowledge, this study is the first to demonstrate that endoscopic APC is an effective and safe treatment option for dumping syndrome with PPH after RYGB during long-term follow-up without pharmacological treatment. In addition, all patients with previous WR experienced significant weight loss during the 2-year follow-up.

## Data Availability

The raw data supporting the conclusions of this article will be made available by the authors, without undue reservation.
